# “Let it be as it is”: between shock and acceptance - emotional, identity, and cognitive responses to the diagnosis of neurofibromatosis type1

**DOI:** 10.1186/s13023-026-04428-w

**Published:** 2026-06-12

**Authors:** Katarzyna Kowal, Jan Domaradzki

**Affiliations:** 1 Wladyslaw Bieganski Collegium Medicum, Jan Dlugosz University in Czestochowa, Aleja Armii Krajowej 13/15, Czestochowa, 42-200 Poland; 2 Department of Social Sciences and Humanities, Poznan University of Medical Sciences, Poznan, Poland

**Keywords:** Diagnosis experience, Neurofibromatosis type 1, Emotional responses, Identity and illness, Meaning-making, Patient perspective, Psychosocial adaptation, Rare diseases

## Abstract

**Background:**

Neurofibromatosis type 1 (NF1) is a rare genetic condition characterised by visible symptoms, clinical uncertainty, and psychosocial complexity. The experience of diagnosis often represents a key turning point that shapes personal identity, relationships, and perceptions of the body. However, little is known about how individuals make sense of and emotionally respond to receiving an NF1 diagnosis. This study aimed to explore the cognitive, emotional, and identity-related dimensions of the diagnostic experience among people living with NF1 in Poland. A qualitative study was conducted using semi-structured, in-depth interviews with 93 adults diagnosed with NF1, and the data were analysed using Reflexive Thematic Analysis.

**Results:**

The findings indicate that receiving an NF1 diagnosis is not a single clinical event but an evolving identity process shaped by time, personal history, and social context. Participants’ reactions to diagnosis varied depending on the diagnostic context and communication process. Four main patterns of response were generated: (1) lack of emotional reaction, relief, and acceptance of the diagnosis; (2) search for knowledge and active engagement with information; (3) denial, minimisation, and emotional distance; and (4) emotional crisis, somatic anxiety, and existential disorientation. The diagnosis also acted as a catalyst for redefining life, the body, and everyday experience, expressed through heightened bodily awareness and a reordering of life priorities, values, and relationships. Together, these themes demonstrate that the NF1 diagnosis functions both as a crisis and a transformative opportunity. It simultaneously confirms embodied experiences and challenges established self-concepts, prompting reflection, self-awareness, and biographical reorientation.

**Conclusions:**

The study highlights the importance of timely psychosocial support, empathetic and clear communication, and family-centred care in the diagnostic process. Understanding the emotional and identity-related responses to diagnosis may inform more holistic clinical practices and improve the quality of care for individuals and families affected by NF1.

## Background

Neurofibromatosis type 1 (NF1) is a multisystem genetic disorder caused by pathogenic variants in the *NF1* gene. It is inherited in an autosomal dominant manner, although approximately 50% of cases result from new, spontaneous mutations [1, 2]. Gene penetrance is nearly complete, but phenotypic expression varies widely among individuals [1]. Typical clinical features include *café-au-lait* macules (CALMs), intertriginous freckling, cutaneous neurofibromas, and learning or behavioural difficulties [1]. NF1 is also associated with an increased risk of developing tumours such as gliomas, malignant peripheral nerve sheath tumours, and several solid malignancies, including breast cancer and leukaemia [3, 4]. Although new targeted therapies are emerging and show promising outcomes, treatment of NF1 remains largely symptomatic [5, 6]. Although individuals with NF1 may have a near-normal life expectancy in the absence of serious complications, population-based studies suggest moderately reduced survival, particularly among individuals with severe disease manifestations, high tumour burden, malignant peripheral nerve sheath tumours, or other serious complications, although estimates vary considerably across studies and clinical subgroups [7–9].

The first diagnostic criteria for NF1 were developed in 1988. They were based on a set of characteristic clinical features, including pigmentary changes, iris Lisch nodules, skeletal abnormalities, optic pathway gliomas, and a positive family history [10]. In 2021, an updated version of the criteria was published, allowing confirmation of the diagnosis through genetic testing and incorporating new clinical indicators such as choroidal abnormalities, revised definitions of osseous lesions, and reclassified optic pathway gliomas [11, 12]. This revision has facilitated earlier recognition of NF1, particularly in children who do not yet meet the full clinical criteria, and has significantly reduced the time between initial presentation and definitive diagnosis [13–16].

Despite these advances, NF1 remains a diagnostically challenging condition. Although the diagnosis can often be made clinically and genetic testing is not always required, awareness of the disorder among clinicians, particularly general practitioners and paediatricians, remains insufficient [17–19]. In Poland, as in many other countries, the diagnostic process for NF1 is frequently prolonged, fragmented, and burdensome, placing significant emotional and financial strain on affected individuals and their families [17, 20–22].

A rare disease diagnosis, such as NF1, is not only a medical event but also a profoundly psychosocial and identity-shaping experience [20, 23–25]. Research shows that receiving information about a genetic condition influences one’s bodily self-perception, social relationships, life plans, reproductive decisions, and everyday functioning, becoming a key biographical turning point [22, 26–29]. Studies also emphasise that the diagnosis of a rare disease is often accompanied by uncertainty, stigma, limited specialist knowledge, and a prolonged “diagnostic odyssey,” all of which place a considerable emotional and practical burden on patients and their families [30, 31].

At the same time, there remains a lack of research describing how individuals with NF1 experience and interpret their diagnosis and what meanings they attribute to it. The impact of an NF1 diagnosis on emotions, identity, body image, social relationships, and everyday functioning is also insufficiently understood. This gap is particularly evident in Poland, where the psychosocial dimensions of living with NF1 remain largely undocumented despite the specific diagnostic and organisational challenges of the healthcare system.

Therefore, the aims of this study were to: (1) examine the cognitive, emotional, and identity-related dimensions of the diagnostic experience; (2) identify distinct patterns of responses to receiving an NF1 diagnosis; and (3) explore how diagnosis becomes a biographical turning point that prompts a redefinition of life, the body, and everyday relationships.

## Material and methods

### Study design

This article presents a focused thematic analysis conducted within a broader qualitative research project conducted by the first author (KK) exploring multiple dimensions of living with neurofibromatosis type 1 (NF1). Previous publications based on this interview cohort examined the altered body and its implications for family interactions and stigma [22], emotional, identity-related, and reproductive dimensions of living with NF1 [29], as well as diagnostic trajectories and experiences of delayed diagnosis [32]. In contrast, the present analysis focuses specifically on the emotional, cognitive, and identity-related dimensions of the diagnostic experience, examining how individuals with NF1 interpret, emotionally respond to, and integrate the diagnosis into their personal biographies and everyday lives. Rather than concentrating primarily on embodied experiences, interpersonal relationships, or diagnostic trajectories, this study examines how individuals with NF1 emotionally process diagnosis, negotiate identity, and reinterpret their bodies, relationships, and everyday lives following diagnostic disclosure. At the same time, although some diagnostic experiences discussed in the present manuscript partially overlap with themes explored in earlier publications, they are interpreted here through a distinct conceptual lens focused on emotional responses, identity reconstruction, and the ways individuals make sense of diagnosis within the context of their everyday lives and biographies.

Given the lack of sociomedical research on this topic, the study was designed as a qualitative one, situated within the constructivist-interpretive paradigm, and employing Reflexive Thematic Analysis (RTA) according to Braun and Clarke’s framework [33–35]. The use of RTA stemmed from its alignment with this theoretical approach and with the descriptive and interpretive aims of the study, enabling the capture of diverse ways of meaning-making in relation to health experiences and the formulation of conclusions relevant to clinical practice.

The empirical basis of the article consists of in-depth individual interviews (IDI) in which participants described: the moment of receiving the diagnosis and its biographical context; earlier assumptions related to family history; the course of the diagnostic process; emotional and cognitive reactions to the diagnosis; the process of becoming aware of one’s own difference; adaptation strategies; the impact of the diagnosis on the relationship with the body and body image; and the consequences of the diagnosis in the areas of procreation, parenthood, and relational and existential functioning. The analysis of these narratives made it possible to capture: (1) the processual nature of the NF1 diagnosis; (2) types of emotional and cognitive reactions to the diagnosis; and (3) redefinitions of life, the body, and everyday life after receiving the diagnosis. The article refers to the authors’ earlier publication on the diagnostic odyssey in NF1, constituting its development and complement by adding the emotional and identity-related perspective on diagnosis.

The study was conducted in accordance with the principles of the 1964 Declaration of Helsinki (2000 revision) and following approval from the Bioethics Committee of Nicolaus Copernicus University in Torun, Ludwik Rydygier Collegium Medicum in Bydgoszcz (decision no. KB 617/2017). All participants provided informed consent before participation and were informed of their right to withdraw from the study at any time.

The study description was prepared in accordance with the consolidated COREQ guidelines [36], which were used exclusively for transparent reporting of the results and not for data collection. Despite criticism regarding their use in studies based on RTA, COREQ remains a widely adopted standard for ensuring transparency and completeness in reporting qualitative health research [37].

### Participants

Participants were selected purposively, with only individuals with a medically confirmed NF1 diagnosis invited to take part in the study. The inclusion criteria were: legal adulthood, informed consent, proficiency in Polish, and access to and ability to use the Internet. Recruitment was conducted in cooperation with two physicians acting as “gatekeepers,” who handed invitations to participate in the study only to patients with a confirmed NF1 diagnosis. In total, 107 individuals received invitations. Recruitment took place at the Oncology Clinic for Phakomatoses of Antoni Jurasz University Hospital No. 1 in Bydgoszcz.

Between April 1 and December 31, 2020, 95 individuals expressed interest in participating in the study by contacting the first author (KK) directly, who also served as the project leader and interviewer. At this stage, she verified in each case whether the individuals had an NF1 diagnosis by asking them several questions about their diagnosis and characteristic symptoms. All applications were positively confirmed. Two individuals withdrew from the study before the interviews were conducted, citing a lack of time as the reason for their withdrawal.

The study included 93 individuals with NF1 (69 women and 24 men). Participants ranged in age from 18 to 64 years, and age at diagnosis varied considerably, from early childhood to adulthood. Detailed characteristics of the study group are presented in Table 1.

**Table 1:** Characteristics of the study group of individuals with NF1

Characteristics of individuals with NF1	
**Gender**	
man woman	24 (26%) 69 (74%)
**Age (in years)**	18–64 years M = 36.69 SD = 10.13
**Age at diagnosis**	1–51 years M = 17.55 SD = 12.18
Diagnosis in childhood (0–12 years) Diagnosis in adolescence (13–18 years) Diagnosis in adulthood (19+)	39 (41.9%) 17 (18.3%) 37 (39.8%)
**Total**	**93 (100%)**

None of the participants had previously known the researcher or had any contact with her before recruitment. The researcher had no personal experience with NF1; her motivation for conducting the study was purely scientific, which was communicated to the participants.

### Data collection

The study was conducted between August 1 and December 31, 2020, by the first author (KK). Due to restrictions related to the COVID-19 pandemic, the interviews were conducted remotely – either by phone or online. Subsequent transcription, anonymisation, and thematic analysis of the interview material continued until April 18, 2021.

During the initial contact, participants were thoroughly informed about the scientific purpose of the study, its procedures, the voluntary nature of participation, and the possibility of withdrawing without giving a reason. They were also informed about the anonymity of the study and the legal obligations regarding data protection, fulfilled by the researcher and the Data Controller (Jan Dlugosz University in Częstochowa). Participation was based on informed consent given verbally; the consent was recorded at the beginning of each interview and noted in the transcript.

Each participant took part in an individual in-depth interview, using open, non-judgmental questions about personal experiences related to NF1 and the meanings attributed to them. Participants were encouraged to speak freely, including on sensitive or intimate topics, with the option to decline answering or to pause the interview in case of emotional difficulty. Most interviews were conducted in the participants’ homes, which fostered a sense of comfort and safety.

The interviews lasted, on average, approximately 90 min and were recorded with the participants’ consent. All recordings were transcribed verbatim based on the audio recordings. In addition to the spoken content, the transcriptions also included paralinguistic elements such as pauses, hesitations, changes in intonation, or emotional nuances in speech, whenever these were relevant for preserving the full communicative and interpretive context.

In total, 134 h, 26 min, and 35 s of audio material were collected. Due to the richness, detail, and diversity of the data, there was no need to conduct supplementary interviews. Participants were informed that they could request access to their transcripts at any stage of the study. None of the participants requested transcript verification or authorisation.

To ensure full anonymity, no data allowing participant identification was collected. Pseudonyms were assigned, and all information that could reveal their identity was removed or modified. Participants were reminded not to provide identifying information about other people. All transcripts were checked for the risk of indirect identification. These procedures were designed to protect the privacy of both the participants and the physicians mentioned in the narratives.

### Data analysis

Although previous analyses based on this interview cohort employed Constructivist Grounded Theory, the present study used Reflexive Thematic Analysis (RTA) following Braun and Clarke [33–35]. This methodological choice reflected the distinct analytical aims of the current paper. While earlier studies focused on developing conceptual understandings of bodily experiences, reproductive decision-making processes, and diagnostic trajectories, the present analysis aimed to explore meanings, emotions, identity-related experiences, and future-oriented interpretations associated with living with NF1. RTA was considered particularly suitable for examining participants’ subjective interpretations and the co-constructed nature of meaning-making surrounding illness, temporality, and uncertainty.

The analytic process followed the six phases proposed by Braun and Clarke [33–35]. The analysis was primarily inductive, while remaining attentive to participants’ explicit accounts and meaning-making processes. In the first stage, the researcher repeatedly read the transcripts, making notes and initial analytical reflections. Next, based on meaningful excerpts related to the experience of an NF1 diagnosis, she developed preliminary, inclusive codes, which were created manually. In the following stage, the codes were refined and grouped into broader interpretive ideas, allowing for the construction of potential themes. The themes were then repeatedly reviewed and modified through a reflexive process involving re-engagement with the data, comparison of codes, and analysis of the relationships between emerging categories.

After refining the boundaries and scope of the themes, they were given final names, and their analytical content was described, emphasising the meanings attributed to the experience of an NF1 diagnosis by the participants. The interpretive process was further developed during the writing of the research report in collaboration with the second author, which allowed for deeper reflexivity and analytical clarity. The researcher also engaged in regular self-reflection on her own interpretive assumptions and positioning throughout the analytic process in order to enhance reflexivity and analytical transparency.

## Results

Three overarching themes with associated subthemes were generated (Table 2): (1) the diagnosis as an identity process; (2) types of emotional and cognitive responses to diagnosis; and (3) the diagnosis as an impetus for redefining life, the body, and everyday experience. Together, these themes illustrate that an NF1 diagnosis represents both a moment of disruption and a biographical turning point – a process of adaptation and meaning-making that transforms personal identity, family roles, and perceptions of the body, encompassing a wide range of emotional, cognitive, and relational responses.

**Table 2:** Themes and subthemes emerging from qualitative analysis

Theme	Subtheme	Types of Reactions / Participant Expressions
1. Diagnosis as an Identity Process	1.1. Normalisation and Integration of Illness within Personal Identity	Acceptance of chronicity, normalisation of symptoms, retrospective meaning-making; treating the condition as part of self and daily life.
	1.2. Growth and Familiarisation with the Diagnosis	Gradual understanding and self-awareness of illness; internalisation through development and age; growing realisation of heritability.
	1.3. The Child’s Diagnosis as a Turning Point in Parental Identity	Neutral attitude toward one’s own illness; prioritising the child’s health; guilt and responsibility; redefinition of parental role.
2. Types of Responses to the Diagnosis	2.1. Lack of Emotional Reaction, Relief, and Acceptance of the Diagnosis	Informational void, indifference, cognitive distance, formal acceptance of diagnosis, relief after years of uncertainty.
	2.2. Search for Knowledge and Active Engagement with the Diagnosis	Curiosity, rationalisation, pragmatic acceptance, information seeking, using the internet, and patient groups for coping.
	2.3. Denial, Minimisation, and Emotional Distance from the Diagnosis	Avoidance, ignorance of medical meaning, downplaying seriousness, fear of progression or malignancy, preferring uncertainty over determinism.
	2.4. Emotional Crisis, Somatic Anxiety, and Existential Disorientation	Shock, despair, guilt, anger, fear of heredity and cancer, withdrawal, body shame, chronic vigilance, “living on a ticking bomb.”
3. Diagnosis as an Impetus for Redefining Life, the Body, and Everyday Experience	3.1. Increased Bodily Awareness and Attentiveness to the Body	Heightened observation of bodily changes, ambivalence toward body image, rational health monitoring, and functional self-care.
	3.2. Reordering of Values and Relationships	Re-evaluation of life priorities, increased empathy, appreciation of health and family, spiritual reflection, hope, and resilience.

### Diagnosis as an identity process

An NF1 diagnosis was not always a turning point. For many participants, it was rather a gradual process integrated with everyday experiences of the body and difference. The identity of a person with the condition was constructed retrospectively, rather than suddenly.

#### Normalisation and integration of illness within personal identity

For many participants, the diagnostic process did not have a clearly remembered starting point. The illness becomes part of everyday life without a reference point, without a distinct beginning in memory. Participants were unable to identify a single moment of recognition, reflecting the gradual path to an NF1 diagnosis, both by physicians and the patient’s family. Before the NF1 diagnosis was pronounced, participants had already experienced a sense of difference.

*I have four sisters*,* two brothers*,* and no one else has this*,* only me. Neither my parents nor grandparents*,* nor anyone else*,* was affected.* (P24)

*No one in the family had been given such a diagnosis. And no one looked like that. No one!* (P30)

Their medically undefined and atypical bodily issues suggested that “something was wrong” (P90). This experience of being “different” (even in terms of body image) constituted a pre-reflective phase of illness awareness, which only gained a name and explanation after the formal diagnosis. The NF1 diagnosis is here understood as a process of negotiation rather than a single event. It serves as a formal confirmation of previously felt intuitions but is not associated with a breakthrough in the patient’s awareness. Acceptance of the NF1 diagnosis occurs naturally, without the need for reflection or reworking one’s life story.

A retrospective interpretation of life with NF1 symptoms occurs. Participants are unable to situate the diagnosis in time, either by age or by a specific temporal framework. However, receiving the diagnosis allows them to begin understanding earlier symptoms and their impact on life (pain, shame, withdrawal). The past is interpreted by participants in the light of medical knowledge. Integration of the diagnosis occurs in a smooth and natural way. Shock or disbelief is rarely a reaction to the NF1 diagnosis. Likewise, there is no fear, dramatization, or terror of the unknown, as the disease is already familiar to the participants.

*I was different from childhood. I mean*,* I looked different*,* developed differently*,* I just formed differently. I don’t even know how to put it. I didn’t develop the same way as my peers. Since childhood*,* I also wore glasses and had vision problems. I had those spots.* (P90)

*When the doctor examined me*,* based on a physical assessment*,* he accurately identified the disease. He told me that this disease is precisely about such changes and that it is incurable. And I actually knew that from the very beginning. (…) Because I did not experience any related ailments*,* the NF1 diagnosis did not provoke any emotional excitement or anxiety in me.* (P25)

The illness is internalised by participants as something they can live with daily. This reflects a strategy of normalising the diagnosis, treating the disease as a part of everyday life that does not completely define the identity and life of the affected person. Rather, the diagnosis is accepted as a natural complement to the existing state. This type of reaction to an NF1 diagnosis demonstrates an effort to consciously come to terms with the illness, accept its presence and consequences, and maintain normalcy in daily life. It allows for living with the disease without being dominated by anxiety about the future.

*I want to live normally. You have to take care of yourself*,* just keep an eye on it*,* and keep living.* (P23)

*Life is too beautiful to dwell on it*,* so you have to accept that you have it. The disease*,* at its current stage*,* let it be.* (P45)

*Before* [the diagnosis], *there was a kind of subconscious acceptance*,* and later it became a conscious one. I reflected*,* analysed*,* and concluded that you cannot give in to obstacles; I tried all the more to be open*,* to enjoy various things*,* and to overcome certain barriers. Somehow*,* more with a smile.* (P8)

#### Growth and familiarisation with the diagnosis

For participants whose diagnosis occurred during infancy or early childhood, the NF1 diagnosis appears as an experience conveyed by others. They do not remember the moment of diagnosis or the first symptoms. They reconstruct their experience through the accounts of parents and physicians, who gradually make the individual aware of their condition. This reflects an initial lack of illness awareness and an indirect understanding of themselves as someone affected. The diagnosis is not fully comprehended at the time it is given. At a young age, participants still hope for a cure and do not fully understand the chronic nature of the disease. Only over time do they come to accept the meaning of the diagnosis. The development of illness self-awareness occurs alongside age progression, leading to a better understanding of the condition. This indicates the processual nature of assimilating the diagnosis. With age and advancing symptoms, awareness of the consequences of the disease increases. Entry into a conscious phase of living with the disease generally occurs during adolescence, particularly when reflection arises regarding the implications of the diagnosis, heredity of the disease, and reproductive plans.

*I mainly remember the beginnings from my parents’ stories*,* because I had been ill from a very young age. I was 4 years old when the ophthalmologist made the diagnosis based on Lisch nodules.* (P52)

*My parents started making me aware of NF1 when I was a bit older. Although already in primary school*,* my parents and doctors said that it was an oncological disease*,* and so on. So I guess it just gradually made me aware of the illness.* (P47)

*It was only later that it began to sink in for me that I wouldn’t have children. It somehow started to reach my mind.* (P14)

Sometimes, the diagnosis only becomes real at the moment of a tumour’s malignant transformation and the need for invasive treatment. This moment proves to be a turning point in the patient’s understanding of what NF1 is.

*The moment I became aware of the illness was when I received a referral for chemotherapy and had to wait two months for the results comparison. Because a tumour appeared on the magnetic resonance imaging.* (P52)

#### The child’s diagnosis as a turning point in parental identity

For some participants, a medically confirmed NF1 diagnosis is made when their child is diagnosed with the condition. In such cases, the child’s diagnosis becomes a biographical turning point in how they experience their own illness. There is no reorganisation of personal identity or fear for themselves. From that moment, participants “put themselves aside” (P32) and focus on their child’s disease. The child’s health and future become the priority. Their own illness is treated as “part of fate” (P67), rather than a catastrophe.

*When my son was admitted to the hospital*,* he was nine months old*,* and the disease was diagnosed in him; and we received a referral to a genetic clinic. That’s when I also received the diagnosis. But I put myself aside and focused on my son.* (P32)

*I reacted normally. I somehow accepted it indifferently. I didn’t start worrying because I focused on my daughter*,* who had more serious problems. And I… I’m just like that.* (P44)

*Honestly*,* since I learned about this disease because it was diagnosed in my son*,* I focused entirely on him. On how he would live*,* what would happen if my wife or I were gone*,* and on the financial side. This disease changed my approach*,* reevaluated everything*,* and pushed my own happiness to the background. There is only constant worrying about the quality of life of my son*,* not my own*,* because in such situations*,* you don’t think about yourself*,* only about the child.* (P29)

The strategy of this group of participants is neutrality toward their own diagnosis and a focus on the child. The child’s diagnosis evokes strong emotions – disappointment, guilt. Their own diagnosis is overshadowed by the child’s suffering. It does not trigger a crisis but is accepted as information, which may indicate a defence mechanism developed through years of coming to terms with symptoms based on their own experiences. In the face of an NF1 diagnosis, roles are redefined – the parent primarily sees themselves as the caregiver of a sick child, and only secondarily as a person with the disease/patient.

### Types of responses to the diagnosis

The diagnosis of a rare genetic disease such as NF1 affects the lives of participants on multiple levels: emotional, physical, social, and existential. This is illustrated by the highly diverse reactions of participants to the NF1 diagnosis described below, which depend on numerous variables.

#### Lack of emotional reaction, relief, and acceptance of the diagnosis

The initial lack of an emotional reaction to an NF1 diagnosis results from the patient’s informational isolation, which is often typical for rare diseases, and may result from the inadequate information from physicians at the time of diagnosis. The moment of diagnosis appears here as a formality without support. For some participants, a lack of knowledge about the disease at the outset reduces the sense of threat. A diagnosis given without informational and emotional support does not fulfil its function – it does not lead to understanding the patient’s situation. Instead, disorientation, denial, or ignorance of real risks occurs. There is a “just moving on” attitude (P7). The patient is unconcerned about the diagnosis, perceiving it as a meaningless label. The diagnosis does not become a turning point in their life, nor does it affect their identity or sense of being ill. There is no redefinition of self in light of the diagnosis, nor is there an immediate, deep understanding of the nature of neurofibromatosis. For patients experiencing this situation, comprehension of the diagnosis emerges later, during adolescence, alongside the progression of symptoms, increased information obtained from the Internet, and more frequent contact with specialists.

*No one explained to me exactly what kind of disease it is or what it involves. Only later*,* when I started looking on the Internet and going to the hospital in* [city name], *did I find out exactly what the disease is. At the beginning*,* I didn’t know what it was. That’s why I reacted to the diagnosis normally; I didn’t feel sick*,* I didn’t get examined*,* and I didn’t take it easy at work. I didn’t even know what it was at the time.* (P10)

*There was no reaction; I wasn’t in shock. At that time*,* there were neither books available on this disease nor the Internet. It was unclear what had happened. The doctor said it was a damaged gene and that it was a disease present for seven generations back. As a teenager*,* a 15-year-old girl*,* that didn’t mean anything to me.* (P33)

*No one fully told me what it involved. I didn’t know for a long time what kind of disease it was. The doctor only said that there were tumours on the spine and nothing could be done*,* and that was it. That was the information I initially received. For a long time*,* I didn’t know what it was about or what it could entail.* (P27)

It sometimes happened that participants received a diagnosis presented by the physician using the eponymous name of the disease: “von Recklinghausen’s disease,” which patients did not associate with NF1. The experience of informational chaos was common among patients who received fragmented and incomplete information and, therefore, did not fully understand their diagnosis. It was only through independently searching for information on the Internet that patients learned that the term “von Recklinghausen’s disease” is a historical name for neurofibromatosis type 1 (NF1).

*My parents knew that these spots were not just a feature of my appearance*,* but a sign of the disease. Although in reality*,* no one explained to my parents exactly what it meant. No one really explained to them that scoliosis could result from nodules in the spine (…). Then the Internet appeared*,* and I typed in some keywords. “Phakomatoses and Recklinghausen’s Disease Clinic” came up. I thought*,* “That’s what I have”*,* and I started to look into it. Later*,* I came across an article called: “The World in the Color of Coffee with Milk”. I looked at the diagnostic criteria*,* that there should be five or six spots*,* and I was right on the borderline. I have exactly five; they appear occasionally*,* or they were there*,* and I only noticed them later. (…) They exist and may increase in number*,* I learned that from the article.* (P1)

In situations where the patient or their caregivers have actively and persistently sought a diagnosis, the recognition of NF1 brings relief and psychological calm, although often mixed with feelings of regret and frustration due to incorrect interpretations by physicians or previous shortcomings in the healthcare system. Another ambivalent reaction among participants is that, on one hand, they experience relief from learning the cause of their condition, and on the other, disappointment that it is a genetic disease and not, for example, “just ordinary swelling” (P39). Relief is intertwined here with sadness, arising from the confrontation with the disease’s irreversibility and heritability. It is worth emphasising that even in this case, the medical diagnosis primarily serves a legitimising function rather than a cognitive or explanatory one.

*It was both relief and resignation at the same time. For 29 years*,* the problem for me had been the lymphatic swelling*,* and that’s really what all my search for a solution focused on. Learning that it was actually a genetic defect*,* I thought it would have been better if it were just lymphatic swelling*,* and not a genetic defect*,* which additionally brings certain consequences*,* preventive tests*,* and family planning.* (P39)

For this group of participants, the diagnosis also does not bring a significant change in self-perception. NF1 was already known before the formal diagnosis through the symptoms they had experienced since childhood (spots, nodules) and had become accustomed to. Therefore, the formal recognition did not provoke shock or a sense of a turning point. It is merely a confirmation of what the patient already knows and allows for a better understanding of previous symptoms. This indicates a gradual acquisition of knowledge and awareness of the disease before the official diagnosis. The diagnosis is accepted calmly, without dramatisation, consciously rejecting a “victim of fate” narrative (P4). There is a rationalisation and reconciliation with the diagnosis, which is treated as something “that was already known anyway” (P88), especially if follow-up examinations currently show no health threats and the symptoms are being monitored but remain stable.

*I’ve lived my whole life with this disease without knowing about it*,* and nothing really happened to me. And nothing is happening now. I just have these spots*,* and nothing is happening. I had a brain magnetic resonance imaging*,* and nothing showed up. I’m not freaking out about my disease.* (P3)

*And generally*,* nothing is happening*,* nothing concerning that could threaten my life or affect any internal organs.* (P23)

*I’m not making a tragedy out of it. It happened to me*,* well*,* okay*,* I have to live with it.* (P4)

*It wasn’t a big surprise for me. I already knew that I had it.* (P34)

A late NF1 diagnosis does not trigger an existential breakthrough. It confirms previous suspicions but does not cause a fundamental lifestyle change. It can be assumed that as long as the symptoms do not significantly affect the participants’ daily functioning, the NF1 diagnosis will not be experienced as traumatic.

#### Search for knowledge and active engagement with the diagnosis

The reaction to an NF1 diagnosis presented without an explanation of the nature of the disease is the awakening of a need in the patient to acquire knowledge independently. The diagnosis is perceived as unfamiliar and vague. The term “NF1” did not provoke significant anxiety because patients did not have access to knowledge about this rare and little-known genetic disorder. In the absence of informational support and patient education, the Internet becomes a tool compensating for systemic deficits and serves as a source of support in emotionally processing the diagnosis. Initially, online information searches are superficial, but later they become intensive and systematic. Through the Internet, participants also encountered the difficult realities of other patients, especially those with more severe courses of the disease, which they came across by visiting patient association websites and social media platforms.

*At first*,* I didn’t know what it was. I didn’t understand what I was reading on the Internet. I was typing in what the doctor had told me.* (P24)

*Since the Internet became available*,* I started using it and started reading. I also began looking for people with this disease. The people I communicated with said that it was hard for them*,* that they were broken.* (P16)

*When I turned 16*,* I became aware of the disease. I was looking for various information on the Internet*,* including the* [name of the association], *which I have been following for a long time. (…) Later*,* of course*,* different platforms like Facebook as well. You could get every opinion about your diagnosis*,* compare things*,* and so on. (…) I also simply started searching a lot about genetic diseases.* (P37)

The type of reaction to an NF1 diagnosis discussed here is characterised by a lack of dramatisation, replaced instead by pragmatism and rationalisation. Information seeking is an action aimed at organising knowledge about the disease, driven by the need to control the situation. The disease begins to appear as an object of cognitive fascination – the patient reveals a strong desire to learn about NF1 and actively searches for new information. This is a deliberate adaptive strategy of the patient.

Consciously accepting the diagnosis meant, for participants, an awareness of the threat posed by the disease and the necessity of symptomatic treatment to mitigate its effects and prevent complications. Patients responding to the diagnosis in this way were aware of the seriousness of the situation as well as the lack of alternatives. The diagnosis imposed the need to remain under the care of specialists, in which participants perceived no room for choice. This type of reaction to the diagnosis is strongly associated with the experience of losing individual control over one’s own body.

*Experiencing the diagnosis was a difficult moment for me. Then*,* immediately*,* there was a brain tumour surgery. In an instant*,* upon reading the diagnosis*,* my whole life changed. I knew it was a serious operation; I knew that the surgery had to happen.* (P1)

*As for the diagnosis of this lymphatic swelling*,* I always had tests done*,* which showed that my lymphatic system was functioning normally. (…) And having already known that it is a genetic disease*,* I knew that it could be a plexiform-type lesion.* (P39)

The diagnosis provides the possibility of naming the problem and changing one’s approach to their own body. It facilitates the understanding of symptoms. From the moment of diagnosis, patients undergo preventive examinations and consciously plan for the future (e.g., in the context of offspring).

#### Denial, minimisation, and emotional distance from the diagnosis

Another type of reaction to the diagnosis is a strategy of denial or minimisation, in which participants tried not to worry about the disease. Since NF1 is a condition with uneven progression, this reaction is supported by periods of plateau, during which clinical symptoms remain relatively stable. This type of reaction also occurs when the person with NF1 has a limited understanding of the disease’s consequences. There is a strong focus on the present, and the topic of future disease progression is suppressed. The diagnosis may be understood here as an empty, formal label that does not affect reality. The diagnosis has semantic, rather than existential, significance. It is overshadowed by the experience of one’s own body. For these participants, the body and its symptoms form the basis of knowledge about the disease, not the medical label. The diagnosis does not influence their daily life as long as pain or functional limitations do not appear. The disease is experienced more through the senses than through the words of doctors.

*It doesn’t bother me. I have a few spots on my stomach. It doesn’t interfere with my daily life or work at all. I feel as if I were completely healthy. Nothing is happening. Absolutely nothing. I’m not struggling or anything. Nothing.* (P20)

The strategy of minimisation and denial, however, proves ineffective when the disease worsens. The appearance of new symptoms forces a reinterpretation of the diagnosis and a radical change in the approach to NF1. The diagnosis gains new meaning, becoming real and embodied.

*The doctors said it was Recklinghausen’s disease. (…) But I didn’t really take it to heart*,* because they said that’s what it was*,* so I never looked into it. It didn’t hurt me*,* it didn’t bother me in any way*,* so I didn’t explore it further. Only when I had that episode with the painful stomach did I end up in the hospital*,* and they wanted to remove my kidney*,* my gallbladder*,* and something else*,* but each time I refused*,* because I knew that wasn’t what was causing my pain – that it was something else. It turned out that the nodules were between the vertebrae*,* so the pain was just from that*,* and nothing else.* (P72)

Some participants consciously avoided deepening their knowledge about the disease, especially in the context of a family history of cancer. Emotional and cognitive distancing from the disease generated fears about the development of malignant tumours in the course of NF1. Avoiding information about NF1 as a way to avoid stress and anxiety serves here as a coping strategy, a kind of defence mechanism.

*I started reading about it*,* but I guess I just didn’t want to stress myself out. Because I started reading that there are cases of tumours in NF1. So I preferred not to deepen that knowledge. Especially since my mother died of cancer*,* and my grandmother had cancer.* (P63)

After hearing the NF1 diagnosis, participants also express resistance to accepting it as a definitive verdict on the course of their lives. They prefer unpredictability over genetic determinism and reject deterministic thinking about themselves in the future.

*I think I would have preferred that it didn’t determine everything that could happen to me in life. I think I would have preferred the version where it’s an unpredictable disease*,* so you don’t know what might happen.* (P51)

They try not to panic and to maintain balance, being cautious and following medical recommendations (e.g., avoiding sun exposure) without imposing excessive restrictions. They want to live life to the fullest and do not want the NF1 diagnosis to define them. They do not want their sense of self to be shaped solely by the disease. They want the disease to be just one of many aspects of their lives. They are open to experiencing the disease and all that it may bring in its unpredictability. The quoted participant does not see herself as a sick person in an existential sense. The diagnosis does not disrupt her existing identity but rather complements it in a coexisting manner.

#### Emotional crisis, somatic anxiety, and existential disorientation

In another type of reaction, participants describe the NF1 diagnosis as unpleasant, generating an emotional burden, especially since, for years, they did not know what they were dealing with. Among the emotional reactions reported are feelings of regret and anger toward parents who conveyed the information about the disease briefly or took a passive stance toward its symptoms. According to the participants, this affected the way they later processed their illness-related identity. Above all, participants in this group expressed dissatisfaction with being informed about the disease late and with the lack of support in understanding the diagnosis. After receiving the diagnosis, they were left alone with the information and did not know what to do next.

*My parents just said that I have it*,* and that was it. That they couldn’t change it*,* that it just happened.* (P35)

*It was a shock when I learned about the disease from the doctor. And it’s partly my parents’ fault. They neglected it. They knew about the disease but did nothing. When I asked my mother if she knew anything about it*,* she said she did.* (P93)

Among the emotional reactions to the diagnosis, feelings of helplessness also emerge, along with questions such as: “Why me?” Participants express incomprehension and a sense of unfairness.

*I was simply very scared. Somehow*,* I just couldn’t wrap my head around it. I kept saying: Why me*,* why my son?* (P32)

*It was really hard. I kept asking why it had to be me. I couldn’t come to terms with it for a long time.* (P41)

The NF1 diagnosis is an event that triggers an emotional crisis. Participants experience disappointment and helplessness in the face of the disease’s irreversibility and lack of treatment. The diagnosis does not bring relief; on the contrary, it becomes a source of tension. Awareness of the disease does not reduce suffering; rather, it becomes a burden. There is a sense of being overwhelmed by the risk of passing the disease on to children and the obligation of constant monitoring.

*I felt such disappointment when I learned that it is an incurable disease*,* and that it also has consequences when it comes to starting a family and wanting to have children.* (P8)

*The awareness that I am seriously ill only makes it worse. (…) If it were possible*,* I think I would rather not know that I am ill. Maybe it would be easier.* (P36)

In response to learning their NF1 diagnosis, these patients limit social contacts, even within their families. Withdrawal from previous activities, social retreat, self-isolation, and a reduction in relationships occur for various reasons. One of these is low self-esteem, which gives rise to a fear of stigmatisation. Awareness of the disease increases with age. While as children participants were not conscious of the disease, with age and progression, the disease increasingly becomes a source of embarrassment.

*I even stopped visiting my family and constantly explaining what the results were and everything. Honestly*,* I just don’t have the energy or desire for that.* (P29)

*I became a more closed-off person when it comes to other relationships and showing myself*,* my body. It embarrasses me more now. I wasn’t aware of it before. I’m afraid that if I told newly met people*,* they might treat me as an inferior person. This affects my relationships with others and my willingness to open up to them.* (P35)

When the NF1 diagnosis is made in the context of an acute disease episode (e.g., optic nerve glioma, malignant tumour), the reaction to the diagnosis is most often emotional shock, followed by despair, inner collapse, and intense anxiety. A common form of adaptation to the diagnosis in such cases is the immediate and much-needed release of emotions.

*It was just like a blow. Of course*,* I cried for several nights after finding out that I had something like this.* (P11)

*I was not yet eighteen*,* I collected the result*,* I was waiting in line for the neurologist*,* and I was reading the diagnosis. I remember that the first thing was that I got scared and I cried.* (P2)

*When I found out*,* it was a disaster for me. I cried*,* I didn’t want to go to school. I thought that my whole life had collapsed.* (P1)

The diagnosis appears as a starting point for long-term emotional processing of the disease. Over time, concerns emerge about appearance, anger, and fear of worsening health due to the increased risk of developing cancers, such as breast cancer or leukaemia, or the threat of disability caused by an unfavourable tumour location. As patients gain greater awareness of the disease’s consequences, anxiety arises regarding an uncertain future, chances of survival, and the possibility of passing the disease on to their children.

*The worst moment was probably when we once went to a medical symposium and the doctors were talking about the diseases we are more likely to develop than a normal*,* healthy person. And I remember they mentioned leukaemia*,* among others*,* and I got really scared then.* (P13)

*It still brings up emotions. Sometimes it’s mentally very hard*,* especially when the results are bad or when they don’t really know what’s going on. These emotions are always with me*,* and I get angry about it.* (P47)

*I’m worried right now*,* and I don’t know what will happen next*,* whether the tumour will keep growing or not.* (P22)

*When I found out what this disease was*,* I didn’t know what to expect. Nobody knew anything.* (P1)

Interestingly, this anxiety sometimes took the form of somatic fear, while patients remained cognitively indifferent to the NF1 diagnosis itself and its content. What frightened them were the symptoms – deteriorating physical condition, such as hand weakness, difficulty writing, or walking. Patients were also concerned about the possibility of visible changes appearing on the face. The somatic fear of the unknown is also related to not knowing what was happening inside the body. The uncertainty associated with the high somatic expression of symptoms was described as “living on a ticking time bomb” (P19).

*I want these changes on my face to appear as late as possible*,* or ideally not appear on my face at all. (…) I assume that internally I am safe*,* but that’s just an assumption. I know that new ones will keep appearing*,* because they constantly do*,* all sorts of them. I’m afraid they might affect my optic nerve*,* the inner ear*,* or my spine.* (P42)

This type of reaction can be considered a typical defensive response to the diagnosis – the patient distances themselves from the difficult information (the diagnosis) while simultaneously experiencing strong anxiety about more tangible aspects, namely the symptoms. Another manifestation of distancing from the diagnosis is the inability to recall the year it was given during a biographical narrative. Among these participants, a difficulty in situating this event in time was identified.

### Diagnosis as an impetus for redefining life, the body, and everyday experience

The NF1 diagnosis served as a starting point for transformations both in the perception of one’s own body and in the re-evaluation of life and social relationships. For the participants, it represented both a source of crisis and an impetus for reflection, a shift in priorities, and the construction of a new sense of life.

#### Increased bodily awareness and attentiveness to the body

The NF1 diagnosis did not change the participants’ relationship with their own bodies, which had generally already been negative or ambivalent. There was a persistent distance from the body, which had long been a source of low self-esteem, shame, and non-acceptance. In this context, the diagnosis did not bring relief; rather, it heightened awareness of the burden of the disease.

*Absolutely*,* the diagnosis does not change one’s attitude toward the body in terms of appearance. I don’t like my own body; I would get rid of my own body.* (P51)

*When it comes to accepting my own body*,* I have always had low self‑esteem. These are illness‑related complexes. (…) As for the surgery*,* they had to shave off part of my hair*,* and that really got me down*,* even though I have thick hair. I felt like something was simply missing.* (P2)

*I always tried not to worry about how I look. Although maybe I did not want to worry*,* I was still experiencing it internally.* (P14)

Since the body had already been a source of symptoms and anxiety, the diagnostic label itself does not introduce a new quality. In none of the participants was a situation observed in which the diagnosis improved self‑acceptance. It does not lead to liking one’s body, although it also does not significantly deepen the body‑image crisis. What is present, however, is a constant body orientation and a problem with self‑acceptance due to progressing symptoms, and consequently, an aesthetic and social stigma.

*I look like I’ve been beaten up all the time. I simply have a tumour above one eye*,* and I have a spot on one eye and the other*,* and the tumour on my right eye is growing.* (P10)

*The diagnosis was in 2009*,* and back then*,* I didn’t really feel anything like that yet. But during pregnancy*,* those fibromas and neurofibromas started appearing*,* and that’s when I began to look at my body differently*,* with more disgust.* (P2)

Participants report that they try not to dwell on either the diagnosis or the appearance of their bodies, although they also acknowledge experiencing difficult situations. Nevertheless, the diagnosis certainly increases their bodily awareness, helping them to better understand skin changes that were previously completely incomprehensible. Participants gain the understanding that these changes in their bodies are the result of the disease, not their “own fault” (P85). Their attention to their bodies also increases – they begin to observe themselves more carefully, check for nodules, and monitor changes.

*I started to look at my body more closely*,* to see if I had any other nodules anywhere. I examine my hands*,* paying close attention*,* and when I hold them up to the light*,* I can see a nodule.* (P43)

For those patients who receive the diagnosis during a severe course of NF1, the moment of diagnosis is biographically anchored as a significant turning point in their lives, giving meaning to prior bodily experiences. The diagnosis of an optic nerve glioma or a brain tumour is described in participants’ narratives as a boundary moment that divides life into “before” and “after” the diagnosis. It disrupts previous stability and sense of security. This is an experience of immediate existential transformation. In this context, the diagnosis becomes a biographical starting point for change and a deeper relationship with oneself. However, participants emphasised that it is not the diagnosis itself that triggers this change. It serves as an important starting point, but genuine transformation in the perception of the disease and of oneself occurs alongside the progression of symptoms.

*I think my attitude toward my body changed after the diagnosis*,* but not because of the diagnosis itself – rather*,* it was when my tumours began to grow. I have more tumours inside my body.* (P46)

*It wasn’t so much the diagnosis that changed it*,* but the subsequent changes in my body.* (P33)

When the NF1 diagnosis is consciously acknowledged, participants perceive it not merely as a medical label but primarily in terms of its consequences – mandatory, regular check-ups and monitoring, as well as the medicalisation of daily life, which affects lifestyle and planning. They also recognise the need for increased self-care.

*The diagnosis made me think that I need to be more careful*,* stock up on things*,* and deal with a lot of doctors’ appointments. That’s how I interpreted it at the very beginning.* (P52)

*I thought that I now have to take better care of myself*,* start going to the doctor – that was my first thought. And generally*,* I have to pay more attention to myself.* (P23)

*Now I know that every year I have check-ups: a magnetic resonance imaging scan once a year*,* blood tests*,* various ultrasounds – I have to keep track of all of it.* (P36)

The diagnosis increases health awareness, the need for systematic medical supervision, and a sense of being “under scrutiny”. It heightens vigilance toward the body and emerging changes. Lifestyle changes are significant and based on a rational approach to the illness. This stance is dominated by pragmatism, along with emotional distance from the disease.

#### Reordering of values and relationships

The NF1 diagnosis influences patients’ approach to the meaning of life, material goods, social relationships, and religion. As a result of reflection on faith and the meaning of life, a re-evaluation of priorities takes place – from material things toward spiritual and family values. An NF1 diagnosis changes the way life is perceived, enabling participants to notice beauty in simple things and to appreciate health more. Hospital experiences, especially observing sick children, change their value system and deepen their empathy and attentiveness.

*I think a person becomes more humble. And when you go around hospitals a bit*,* observe those sick children*,* you start noticing other things and finding joy in different things. And you become a slightly different person (…). I truly enjoy every smile*,* every moment with my child*,* every hug. All of it is just wonderful.* (P43)

The participants perceive their sense of self-worth as a psychological resource that has not been destroyed by the illness, despite its impact on memory and cognitive functions.

*I simply re-evaluate certain things. My attitude towards faith has changed*,* my attitude towards having things has changed. Fewer things make me happy now. Very few material things*,* really.* (P29)

*And I have a strong sense of self-worth. I just do. Despite my imperfections. I have strong self-awareness. I respect myself – that’s what you call healthy egoism.* (P28)

Understanding what an NF1 diagnosis means directs the actions of the patient. For the respondents, the experience of diagnosis initiated greater reflexivity, maturity, and inner mobilisation. It becomes a moment of redefining oneself, one’s actions, and reshaping everyday life and identity. It shows the diagnosis as not only a moment of crisis, but also a potentially developmental one. For some, diagnosis opens a new chapter in life, which is transformed and gains a new meaning.

*It caused me to start developing in the direction I want*,* doing what I like. And maybe that gives me a sense of value. Yes*,* I think that’s how it works.* (P47)

*That’s when I got into social work. I got into helping others and devoted myself to it. I redirected myself into being good to others*,* into being a kind of shield.* (P30)

In situations where the diagnosis is hidden from the patient for many years by parents or caregivers, the reaction to its disclosure turns out to be the awakening of hope as an emotional resource, an increase in motivation to fight for life, and a change in thinking about the future. The diagnosis brings hope rather than breakdown. For a long time, the diagnosis had been more of the parents’ experience than the sick person’s, which later influenced the understanding of NF1 as something external. As children, these participants also did not experience dramatisation from their parents, which allowed them to avoid identity-related trauma.

*I didn’t feel it at all; my parents didn’t make me feel what it was*,* what it could be*,* and what it might involve.* (P42)

*I found out that I could have surgery and that there was a chance things would be better. I simply gained more hope that I could live*,* even if in a wheelchair (…). I just knew I would manage.* (P28)

## Discussion

The findings of this study demonstrate that receiving an NF1 diagnosis is a complex, multidimensional process that unfolds over time rather than a single, clearly defined moment or event. While for some individuals NF1 diagnosis represents a sudden turning point, for many others it becomes an element integrated into the ongoing process of understanding their bodies and lives. Yet, the moment of disclosure often occurs amid informational and emotional insufficiency, compelling patients to seek meaning on their own. While for some the diagnosis triggers shock, fear, and existential distress, for others it brings relief, acceptance, or motivation for growth. Hence, by showing that individuals with NF1 frequently negotiate their diagnosis as both a medical and biographical event, simultaneously disruptive and transformative, our study yielded several important findings.

Firstly, this study suggests that for many participants, NF1 diagnosis became part of an ongoing identity process. Rather than representing a rupture or the beginning of illness, the diagnosis often confirmed what had long been sensed and embodied. Particularly among individuals with visible symptoms since childhood, a formal diagnosis merely provided language for an already lived experience. The process of integration was gradual, involving normalisation, acceptance, and adaptation. Over time, NF1 became one aspect of identity rather than its defining feature [22, 29].

These findings confirm previous studies showing that receiving genetic information often reshapes personal identity, producing a liminal position “between health and disease” [20, 23]. Even results of uncertain significance can be experienced as a “full-blown diagnosis,” producing lasting emotional and behavioural effects such as the “patient-in-waiting” identity [38]. Likewise, Adler et al. describe genetic disclosure as a moment of “identity theft”, disrupting coherence and agency as individuals reconstruct the self in biomedical terms [24]. NF1 participants enrolled in our study similarly experienced diagnosis as both recognition and rupture, requiring renegotiation of self-concepts, relationships, and bodily boundaries. However, this should not surprise, since studies on rare diseases show that diagnosis both relieves uncertainty and imposes a new biomedical identity, demanding continuous negotiation between patienthood and normality [23, 25, 39]. Diagnosis legitimises prior suffering yet introduces new forms of vulnerability and difference, shaping self-perception from childhood onward. In this sense, individuals with NF1, like others with rare genetic disorders, experience identity formation as a tension between normality and difference – between belonging and distinctiveness [23, 25, 39].

This dynamic can be understood through the concept of “geneticization of identity,” where genetic knowledge becomes central to how individuals perceive themselves and their bodies [40]. In the case of NF1, this is particularly pronounced since the condition is visible, hereditary, and progressive, forcing people to negotiate selfhood between normality and otherness [22, 23, 27, 41, 42]. As shown in previous studies, NF1 is deeply anchored in one’s biography and the lived body, where visibility and heredity shape self-definition and identity [23, 27, 29, 41]. The diagnosis offers both a biomedical explanation and a social labelling [42, 43]. Thus, diagnosis often becomes a moment of genetic self-recognition, when embodied experience is reframed through biomedical language that confers both meaning and legitimacy.

In this context, the genetic body becomes a central axis around which people construct their sense of self, stability, and a sense of belonging. Thus, while in late modernity traditional identity anchors such as religion, family, or social class have weakened [44, 45], the “molecular optics” of modern genetics [46] increasingly provides frameworks for self-understanding and self-determination and enables people to manage both surface and interior of the body, shaping what Rose [47] terms the “somatic individual”. Thus, access to genetic information redefines how people experience their bodies and lives, providing both knowledge and control. In this sense, diagnosis can function as a tool of self-knowledge, transforming uncertainty into meaning [40, 48]. In fact, for those with genetic conditions, including NF1, biomedical language serves as both an explanatory framework and a symbolic resource for making sense of bodily difference and life trajectory [22, 24, 25, 29]. This creates what Rose [47] calls a “new molecular ontology of life”, where biological and personal identity merge.

This helps explain why, for many participants, diagnosis was not experienced as the beginning of illness but rather a moment that organised and legitimised prior experiences. It also illustrates how individuals with NF1 integrate their condition into self-narratives, transforming stigma into self-knowledge and continuity [20, 29]. This finding also highlights that in chronic and genetic conditions, the boundary between “being healthy” and “being ill” often dissolves; identity work begins long before formal diagnosis.

Secondly, NF1 diagnosis frequently occurs amid uncertainty and limited communication. Many participants described encounters as brief, impersonal, and lacking explanation or emotional support. Disclosure was often reduced to a label without counselling. Consequently, initially, many responded with indifference or minimisation, only later seeking understanding through self-education and online research. These experiences mirror previous NF1 studies, which describe diagnostic journeys marked by long delays, miscommunication, and limited clinical knowledge [22, 29, 42, 49, 50]. In these studies, participants recalled being told that their skin lesions were “just their nature,” echoing earlier accounts of normalisation [20, 27]. Such dismissive attitudes caused confusion, self-doubt, and mistrust toward professionals, prompting families to become their own advocates [22]. These findings echo analyses of knowledge asymmetry in clinical encounters, where biomedical authority can overshadow patients’ experiential understanding and reinforce dependence on professional interpretation [51–53].

This confirms observations made by others emphasising the diagnostic odyssey – a prolonged, fragmented, and emotionally exhausting process marked by uncertainty, miscommunication, and systemic gaps [30, 31]. Parents and patients often navigate multiple specialists and repeated tests, and inconsistent information before receiving a definitive diagnosis, which leads to frustration, anxiety, and loss of trust in healthcare systems [25]. Poorly communicated results or abrupt disclosure amplify emotional distress [50, 54]. Families describe pre-diagnostic life as a “rollercoaster” of hope and despair, ending in mixed emotions, validation coupled with fear and disorientation [30, 55]. Such uncertainty erodes trust [31], and the absence of communication channels deepens confusion and isolation [56].

Importantly, participants stressed how the quality of communication shaped their experience. Similarly, Burbury et al. [57] show that genetic results, diagnostic or inconclusive, delivered without empathy can be “devastating”, while compassionate, family-centred communication fosters long-term trust [54]. Many families lacked access to genetic counselling or peer support [49, 50]. These patterns reveal the need to address both structural and interpersonal dimensions of the diagnostic process [49]. Previous studies show that systemic shortcomings, i.e., limited clinician awareness, lack of NF1-specific training, and regional disparities, compound distress [27, 29, 42, 54, 58]. Evidence supports training clinicians in rare-disease literacy, empathetic communication, and psychological awareness [30, 31, 57]. Providing continuous counselling, clear explanations, and opportunities for dialogue can mitigate trauma and facilitate adaptation [42, 49].

Thirdly, this study reveals that NF1 diagnosis often constitutes an existential and emotional crisis. For some individuals, particularly those learning of their condition after severe symptoms or malignant complications, it was a moment of profound emotional disruption or even existential rupture. Feelings of shock, despair, and irreversibility were common, intensified by awareness of chronicity and absence of cure. Participants described feelings of helplessness, loss of control, and anxiety about the future, both their own and their children’s. This aligns with Goffman’s [59] and Scambler’s [60] notion of “spoiled identity,” a disruption of self-concept through stigmatising or irreversible information.

Similar identity challenges are documented in other genetic conditions. Klitzman [61] found that individuals at hereditary risk ask: “*Am I my genes?*”, struggling to reconcile biomedical and personal identities. People with NF1 likewise navigate visibility, stigma, and self-acceptance, which reshapes family relationships and self-perception [22]. De Moraes et al. [62] describe NF1 diagnosis as an emotional turning point – a moment redefining daily life and relationships, as patients described their bodies as “ticking time bombs,” symbolising both fear, vigilance, and vulnerability that profoundly shape identity and daily decision-making.

These findings also correspond with other NF1 studies that reveal the emotional and psychosocial weight of diagnosis, especially when patients confront visible disfigurement, progressive symptoms, and genetic uncertainty [20, 27]. The NF1 diagnosis thus exposes individuals to the moral and existential gravity of genetic knowledge, compelling them to renegotiate the boundaries between fate, responsibility, and selfhood.

An additional dimension of this emotional burden concerned the experience of ongoing imaging-based surveillance and repeated medical monitoring. Participants frequently referred to magnetic resonance imaging, biopsies, follow-up examinations, and waiting for results as emotionally significant moments shaping their experience of NF1. For some individuals, imaging findings represented a turning point in recognising the seriousness and unpredictability of the disease, particularly when scans revealed tumour progression or prompted referral for oncological treatment. For others, stable MRI results temporarily reduced anxiety, provided reassurance, and reinforced a sense of control and disease stability. At the same time, waiting for imaging results and confronting uncertain or poorly explained findings often generated emotional tension, anticipatory fear, and compulsive information-seeking. Repeated imaging procedures also became routinised and incorporated into everyday life, contributing to heightened bodily vigilance and constant self-monitoring. These experiences resemble what recent literature describes as “scanxiety” – anxiety associated with undergoing scans and waiting for results [63–66]. Recent studies further suggest that emotional responses to imaging are shaped not only by fear of disease progression but also by the way results are communicated, interpreted, and accessed by patients [67]. Although scanxiety has primarily been explored in oncology, our findings suggest that similar mechanisms of anticipatory anxiety, uncertainty, emotional regulation, and temporary reassurance are also present in chronic genetic conditions requiring lifelong surveillance, including NF1, where repeated imaging becomes not only a clinical necessity but also a psychologically and emotionally meaningful practice shaping how individuals live with uncertainty, risk, and embodied vulnerability.

However, comparable mechanisms emerge in other genetic and neurodevelopmental conditions. In Prader-Willi Syndrome, diagnosis and caregiving trigger long-term family reorganisation and identity reconstruction [28]. In Huntington’s disease, predictive or post-diagnostic disclosure challenges individuals’ sense of self, autonomy, and moral agency [26, 68, 69], producing “ontological uncertainty” – a state in which the future self feels biologically predetermined yet psychologically indeterminate [51, 61]. These processes reshape selfhood and family relations, generating identity negotiation and intergenerational tension, and ultimately transform how people see themselves, their futures, and their moral and existential possibilities [68, 70, 71]. Thus, these findings highlight the need for sustained psychological and psychosocial support from the moment of disclosure and call for comparative research on the psychosocial dimensions of genetic diagnosis.

Finally, this study shows that, despite its disruptive potential, an NF1 diagnosis can also serve as a source of meaning and reflection, acting as a catalyst for transformation, personal development, and growth. Many participants described learning about NF1 as a moment of meaning-making that initiated self-reflection, re-evaluation of priorities, and strengthening of emotional resilience. The diagnosis often inspired greater self-care, lifestyle adjustments, and engagement in social or advocacy activities, providing a renewed sense of direction and purpose.

This transformative potential of diagnosis resonates with Rose’s [47] concept of “biological citizenship,” in which biomedical belonging becomes a form of moral and civic participation. For some participants, awareness of genetic risk became a source of empowerment, a motivation to engage, educate others, and transform genetic knowledge into social capital that converts vulnerability into agency and collective action [46, 47]. These processes echo broader sociological theories of genetic selfhood and moral responsibility, illustrating how individuals integrate biomedical knowledge into narratives of meaning, continuity, and growth [20, 23, 61].

In this context, the NF1 diagnosis was not merely a label but a biographical and moral turning point through which individuals reframed suffering, regained agency, and redefined the self [20, 23, 29, 62]. The awareness of heritability and uncertainty fostered new forms of relationality and care, in which individuals sought to protect, educate, and advocate for others at risk [22, 27, 49]. Biomedical information thus acquires ethical and social significance, shaping both individual identity and collective belonging [46, 47, 61].

Ultimately, the duality of diagnosis, as both crisis and opportunity, captures the dynamic interplay between vulnerability and agency in living with a rare genetic condition [25, 31, 62]. By showing how individuals with NF1 turn biomedical knowledge into a resource for self-understanding and engagement, these findings extend theories of genetic citizenship and selfhood, illuminating how people creatively navigate the boundaries of health, illness, and identity in contemporary genomics [29, 46, 47, 53].

## Limitations

This study offers novel insights into how individuals with NF1 experience and emotionally respond to the moment of diagnosis. Nonetheless, several limitations should be acknowledged. First, although the dataset of 93 interviews is substantial for qualitative research, it is not representative of all individuals with NF1 in Poland. Given the absence of a national rare disease registry, it is difficult to assess whether key subpopulations, such as those with more severe symptoms or limited healthcare access, were adequately captured. Second, the study participants were recruited through two physicians affiliated with a single medical centre, which may have introduced a sampling bias. Individuals outside the public healthcare system or those without regular medical follow-up might be underrepresented. Moreover, because recruitment was conducted through physicians affiliated with a specialist NF1 centre, the sample may overrepresent individuals with more severe or tumour-related manifestations of the disease. This may have contributed to a stronger emphasis on themes related to anxiety, disease progression, and fear of future deterioration. Additionally, the overrepresentation of women in the sample limits the transferability of the findings to male patients, who may have distinct experiences or coping strategies. Third, the study relied exclusively on remote (phone or online) interviews, which, although necessary due to pandemic constraints, restricted the ability to capture non-verbal cues and emotional expressions that often enrich qualitative interpretation. While remote interviews may have increased participants’ sense of privacy and comfort, they also limited the observational depth of the research. Furthermore, all interviews were conducted during a period of intense COVID-19 restrictions in Poland, which may have influenced participants’ emotional experiences and perceptions of uncertainty described throughout the interviews. Fourth, although the interviewees varied in terms of age, educational background, and disease severity, individuals in the later stages of NF1 progression were relatively few. This may have skewed the findings towards earlier or more moderate disease experiences, particularly in relation to emotional adaptation. Finally, all stages of data collection and initial thematic coding were conducted by a single researcher, which could have influenced the thematic emphasis or interpretive framing. While methodological rigour was maintained through audit trails and peer consultation, future studies may benefit from multiple coders to enhance analytical reliability.

Despite these limitations, this is one of the first studies to systematically explore diagnostic reactions among individuals with NF1 in a Central-Eastern European context. It offers a rich and nuanced understanding of how patients make sense of diagnosis, negotiate identity, and cope with uncertainty, findings that may inform psychosocial support models and raise awareness among healthcare professionals.

## Conclusions

Overall, this study demonstrates that the NF1 diagnosis should be understood not as a single clinical event but as a prolonged biographical and social process. Beyond its medical implications, diagnosis constitutes an evolving experience of meaning-making, identity negotiation, and emotional adaptation that reshapes personal and family life. It functions both as a moment of recognition and a biographical turning point, simultaneously confirming embodied experiences and disrupting established self-concepts. While many individuals initially face shock, uncertainty, or lack of information, over time, they often integrate NF1 into their life narratives as one dimension of identity rather than its defining feature. At the same time, systemic limitations, delayed recognition, and inadequate communication amplify uncertainty and emotional burden, underlining the need for early detection, coordinated care, and psychosocial support. The findings highlight the duality of diagnosis as both crisis and opportunity, revealing how individuals transform biomedical knowledge into a source of self-understanding, responsibility, and moral agency.

Based on these findings, several implications can be identified to strengthen support for individuals and families living with NF1:

1. Greater awareness of NF1 among primary-care physicians, paediatricians, and non-specialist clinicians is needed to reduce delayed recognition, fragmented referral pathways, and diagnostic uncertainty. Rare disease education should therefore be more strongly integrated into undergraduate medical curricula and continuing professional development programmes.
2. Early access to genetic counselling and psychosocial support should accompany the diagnostic process, particularly for individuals and families facing uncertainty regarding prognosis, heredity, reproductive decisions, and future life planning. Similar challenges related to reproductive uncertainty and moral responsibility were also identified in our previous study on reproductive decision-making among individuals with NF1.
3. The findings support the further development of coordinated rare disease care pathways within Poland’s National Plan for Rare Diseases (2021–2023; continued for 2024–2025) [72] and specialised structures such as the European Reference Network ERN GENTURIS and national Centres of Expertise for Rare Diseases (OECR). Greater integration between primary care, genetic services, and specialised multidisciplinary centres may help reduce diagnostic delays, fragmented care, and patients’ reliance on self-navigation within the healthcare system.
4. Accessible educational resources, patient organisations, and peer-support networks should be strengthened to reduce informational isolation and reliance on unsupervised internet-based self-education, while supporting adaptation to life with NF1.
5. Future longitudinal and interdisciplinary studies are needed to examine how diagnostic meanings, coping strategies, emotional responses, and identity-related experiences evolve across different stages of the disease trajectory.

## Data Availability

Due to the sensitivity issues, the dataset generated and analysed during this study is not publicly available. However, they may be obtained from the corresponding author upon reasonable request.

## Abbreviations

NF1: Neurofibromatosis type 1
